# Validation and evaluation of a common biomarker in human cancers sera protein detected by a monoclonal antibody UNIVmAb

**DOI:** 10.1186/s13104-019-4780-4

**Published:** 2019-11-14

**Authors:** D. Manjunath, Sunil B. Kumaraswamy, Shashidhar Aladhi Venkatakrishniah, Hitesh Nidumanda Appaiah, Anil Thomas, Shib D. Banerjee

**Affiliations:** 1 Preethi Center of Oncology, Vattavyalil Cancer Trust, Mysore, Karnataka India; 2 Logic and Clue Diagnostics, Mysore, India; 3 Department of Anatomy and Cellular Biology, Tuft University School of Medicine, Boston, MA USA

**Keywords:** ELISA, Western blot, Hyaluronic acid binding protein, H11 (sera antigen), UNIVmAb common biomarker, Cancers sera

## Abstract

**Objective:**

Management and diagnosis of multiple human cancers remains a challenge and search for a common biomarker is still debatable. In this manuscript we have evaluated the use of monoclonal antibody UNIVmAb, to detect the protein (H11) as a common biomarker for all cancers irrespective of the grade and origin. We have shown by both ELISA and Western Blot that the H11 protein, is a unique hyaluronan binding protein that has not been detected earlier. H11 protein was fractionated in an anion exchange column followed by cibacron blue gel exclusion chromatography. Hyaluronan binding H11 protein reacted with Monoclonal antibody UNIVmAb and b-HA inspite of b-Hyaluronan (biotinylated Hyaluronan) interaction and HA-Oligo (Hyaluronan oligosaccharides) competition from various grades of Human cancers sera.

**Results:**

ELISA, Western blot and b-Hyaluronan interactions clearly showed an over-expression of UNIVmAb reacted H11 protein in all fifty cancer’s sera when compared with seventy normal sera. UNIVmAb reactive H11 protein can be used as a common biomarker. We believe, UNIVmAb detected H11 protein, is a unique hyaluronan binding protein, that can be used as a common biomarker for all cancers.

## Introduction

The appearance of cancer serum biomarker is a molecular event that indicates the pathological changes that happen in a particular tissue or cell type during cancer development. The most important part of screening is the ability for an early cancer diagnosis. Cancer diagnosis based on the quantification or localization of a particular antigen in cells, tissues or body fluids [1–5] is a topic of interest with human body fluids (serum) as an important source of minimal invasive biomarkers. The widely used blood test for early detection of cancer of the prostate is PSA (prostate-specific antigen) and the proper use of this test is highly debatable [6]. However, diagnosis using circulatory serum antigens makes the detection of biomarkers more feasible, and it is relatively a non-invasive method to acquire experimental samples [4, 5, 7–11].

The development of monoclonal antibodies (mAb’s) identifying specific antigens expressed by cancer cells offers a diagnostic technique for early cancer detection [12]. mAb’s are widely used reagents in the clinical diagnostic laboratories to develop sensitive immunoassays for the detection of their target antigen in circulation, their levels relate with the disease progression [11, 13–16]. (Drugs/Guidance Compliance RegulatoryI nformation/Guidances CM070107.pdf.

Analysis of human embryonic cells, adult tissues and human serum has revealed the presence of hyaluronan (HA) and its receptors. Hyaluronan is a nonsulfated, high molecular weight glycosaminoglycan consisting of the d-glucuronic acid and *N*-acetyl d-glucosamine [17]. HA is present in the extracellular matrix of most tissues and is enriched in many tumours [17, 18]. It is well documented that HA and its receptors, known as hyaladherins (hyaluronan binding proteins) are involved in matrix regulation, cell proliferation, migration and malignant tumour progression [18]. Hyaladherins not only interact with hyaluronan at the matrix proper but also with hyaluronan in the plasma membrane as cell surface receptors and thus influence cell physiology, including secretion of this protein into the circulatory system [19–21]. Previously, we have identified H11B2C2 clones reactive hyaluronan binding protein and revealed its association with tumour progression by immunohistochemistry [13]. The antigen expression increases during tumour progression irrespective of cancer origin. Antibody produced by the clones, detected the antigen in multiple human cancer tissues/serum and we renamed the antibody UNIVmAb. We assume that UNIVmAb reactive H11 is a protein biomarker in various tumour tissues that may give us crucial diagnostic information in early cancer detection. We have described the procedures for fractionation of serum proteins, mainly hyaluronic acid binding protein (HABP), from various cancer patient’s samples and healthy subjects using single dimension electrophoresis. The detection of H11 antigen using UNIVmAb in multiple cancer’s led us to think that H11 antigen can be a common biomarker for multiple cancers.

## Main text

### Materials and methods

Hyaluronan (Na salt) (Acros Organics, New Jersey, USA). Guanidium hydrochloride, bovine testicular hyaluronidase type I S, EDC [1, ethyl 3-(3-dimethyl aminopropyl) carbodiimide hydrochloride, MES buffer (2-*N*-morpholino ethane sulfonic acid) Protease inhibitors cocktail and biotinylated goat anti-mouse IgG’s HRP conjugate were procured from Sigma chemicals, USA. EZ-Link Biotin LC hydrazide purchased from Pierce, Rockford, USA. Anti-human CD44 (H-CAM, Clone 1M7.8.1) antibody was purchased from Fisher Scientific, USA. Streptavidin-peroxidase conjugate (HRP) obtained from Invitrogen, USA.

### Sample collection and preparation

The study consisted of 70 normal subjects and 50 cancer patients (Lymphoma (2 patient samples), Salivary (1 patient sample), Tongue (4 patient samples), Thyroid (1 patient sample), Buccal Mucosa (1 patient sample), Lung (3 patient samples), Breast (6 patient samples), Stomach (3 patient samples), Gallbladder (1 patient sample), Oesophagus (3 patient samples), Colon (5 patient samples), Pancreas (1 patient sample), Rectal (2 patient samples), Urinary bladder (2 patient samples), Prostate (4 patient samples), ovary (5 patient samples), endometrial (4 patient samples) and cervix (2 patient samples). Serum samples from normal subjects and cancer patients were accessed from cancer hospitals in Mysore, India (Preethi Centre for oncology, and KR Hospital, Logic and Clue Diagnostic center) and the protocols were approved by the ethical review committee (IHEC-UOM NO 35) and the patient’s consent were taken. Blood samples were collected from each patient before any treatment. Samples were centrifuged at 2000×*g* for 30 min at room temperature and the separated sera were stored at − 80 °C. The H&E stained tumour sections of patients were obtained from hospitals and were graded using the TNM grading system. Serum samples treated with 4× lysis buffer, containing 0.2 M Tris–HCl (pH 8.0), 80 mM EDTA, 4 mM PMSF, 4 mM Benzamidine-HCl and 2% Triton X 100 plus protease inhibitor cocktails were centrifuged at 10,000×*g* for 30 min at 4 °C. The supernatant was stored at − 80 °C until further analysis. The protein estimation was done at UV 280 nm and Bradford reagent assay using Bovine serum Albumin (BSA) as standard.

Biotinylated hyaluronic acid was prepared according to Boregowda et al. [13] and Srinivas et al. [24]. In brief, HA dissolved in PBS-A was dialyzed in MES buffer and reacted with biotin_LC-hydrazide and EDC in DMSO. This was Incubated for 16 h and then dialyzed against PBS-A and stored in glycerol at – 20 °C.

### Production of monoclonal antibody UNIVmAb

Hybridoma and the antibody were prepared according to Boregowda et al. [18, 22, 23]. In brief, the hybridoma was grown in DMEM with human serum (pathogen and complement free) that were received from the hospitals. The antibody production in the presence of human serum (any blood groups) did not affect UNIVmAb recogntion of the human H11 antigen. The clones were grown in DMEM containing 10% (v/v) inactivated human serum. After 21 days, the media was collected and precipitated with cold saturated ammonium sulphate solution (final 50%) at 4 °C overnight and centrifuged at 12,000×*g* for 30 min. The pellet was dissolved in PBS and dialyzed against PBS.

### Statistical analysis

Statistical differences between groups from ELISA were analyzed using graphpad prism version 5 software. Results are expressed as the mean ± SD. A diiference with P values is defined as follows: P < 0.001 = extremely significant. For westerblot, image analysis was done using Image J software.

## Methods

### Detection of H11 antigen by ELISA using UNIVmAb

**Fig. 1:**
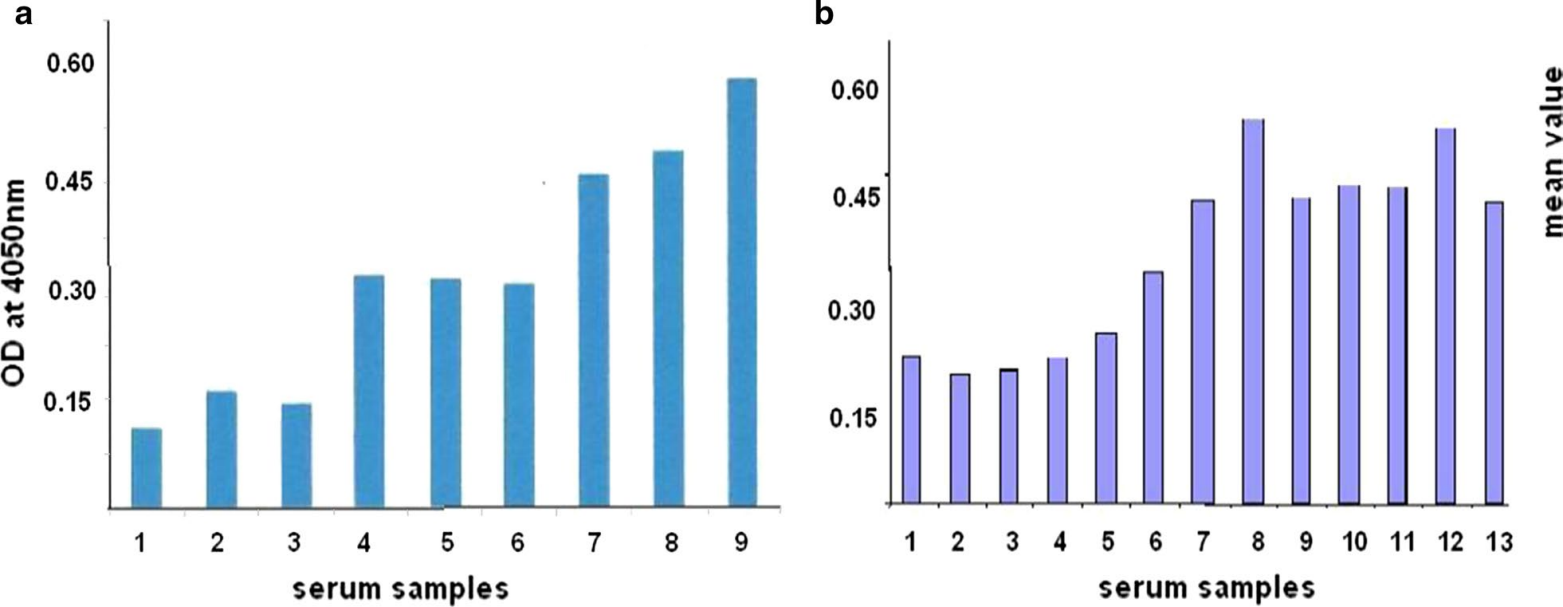
Detection of normal and Cancer antigen by ELISA using UNIVmAb. **a** Lane 1, 2, 3 normal serum (each average of three determinations) Lane 4. Ca stomach Grade 1. Lane 5. Ca tongue Grade 1. Lane 6. Ca Colon Grade 1. Lane 7.Ca stomach Grade 2. Lane 8.Ca cervix Grade 2, Lane 9. Ca Cervix Grade 3. **b** 1–4, normal serum, 5 and 6 Grade 1, Tongue, 7–9 Grade 2, breast, (10–13 Grade 3 samples) 10: Colon, 11: Lung, 12: Oesophagus, 13: Ovary. (average of four samples from each serum). There is gradual over-expression of H11 in sera as the tumour progress

MaxiSorp flat-bottom high protein binding capacity polystyrene-96 well plates were used. Serum samples were diluted with 0.05 M carbonate-bicarbonate buffer pH 9.6 to obtain a final concentration of 1 µg/ml. 100 µl of samples in triplicate were plated on to the 96well plate and incubated overnight at 4 °C. Following day the plate was blocked with skimmed milk (prepared in PBS) for 1 h and incubated with UNIVmAb at 1:10,000 overnight at 4 °C. Following day the plate was washed with 0.2% Tween-PBS followed by incubation with b-goat anti-mouse antibody at 1:20,000 for 1 h and reacted with streptavidin-peroxidase at 1:50,000 for one hour. Plate was washed with 0.2% Tween-PBS and 100 µl of ABTS (1.0 mg/mL) in 0.1 M citrate buffer at pH 4.0 and 5%. Hydrogen peroxide. The reactions were stopped after one hour with 0.2 M citric acid, and the absorbance was measured at 405 nm Fig. 1. Experiments were repeated at least three times. Protein levels were measured by quantitative ELISA.

### Western blot analysis of serum according to Boregowda et al. [15] and Fekry et al. [16]

50 μg proteins from serum lysate were resolved on 10% SDS-PAGE, transferred to PVDF membrane and reacted with UNIVmAb (1:1000 dilution) or anti HCAM mAb (1:1000 dilution) or with bHA probe (1:100 dilution) overnight at 4 °C. Following day, the blot was developed and the proteins were detected using Enhanced ChemiLuminescence (ECL). Since the isolation of antigen is vital to understand its property, we used purified circulating antigen by antibody conjugated CNBR activated Sepharose and Cibacron blue affinity purification method. The purified H11 protein was reacted with UNIVmAb and was also cross reacted with bHA and HA-oligo (500 µg Oligo) competition was also performed. This showed that H11 antigen is a Hyaladherin.

## Results and discussion

We validated the clones H11B2C2 reactive novel HABP in human cancer tissues. HABP overexpression was related to poor tumour outcomes [13, 25]. The antibody produced by the clones detected the antigen in all human cancer tissues and cancers sera; we renamed the antibody as UNIVmAb. We have also identified soluble hyaluronan binding proteins in colon cancer serum with a molecular mass of 57 and 30 kDa [14]. We believed that the soluble 57 kDa H11 protein from colon cancer might have relation with multiple cancer samples. UNIVmAb reactive H11 proteins maybe a new class of hyaluronan binding protein and we did a further investigation in the present study to understand its expression and the nature of H11 protein in various cancer’s sera to further characterize by biochemical analysis and its application as a common cancer biomarker. Table 1 shows the detailed results of H11 expression in various cancers sera.

**Table 1:** Evaluation of UNIVmAb reacted H11 expression in multiple tumours

Tumour types	Grade	Number of the experiment from each patient studied	H11 expression in number of patients in ()
Salivary	I	3	X (1)
Tongue	II	3	Xx (4)
	III	3	xxx
Thyroid (papillary)	II	3	Xx (1)
Buccal mucosa	II	3	Xx (1)
Lung	II	2	Xx (1)
	III	3	Xxx (2)
Breast	I	4	X (2)
	II	3	Xx (2)
	III	4	Xxx (2)
Stomach	II	3	Xx (1)
	III	3	Xxx (2)
Gall bladder	I	3	X (1)
Oesophagus	II	3	Xx (1)
	III	3	Xxx (2)
Colon	I	2	X (1)
	II	3	Xx (2)
	III	4	Xxx (2)
Pancreas	II	3	Xx (1)
Rectum	II	3	Xx (2)
Ovary	II	3	Xx (2)
	III	3	Xxx (3)
Endometrium	I	2	X (1)
	II	3	Xx (2)
	III	4	Xxx (1)
Cervix	II	3	Xx (2)
Urinary bladder	I	3	X (2)
Prostate	I	2	X (2)
	II	3	Xx (2)
Lymphoma	II	3	Xx (2)

We also detected H11 expression from astrocytoma glioblastoma and medulloblastoma not included into the table. XXX = overexpression of H11

*Xxx* very strong; *xx* moderate; *x* weak

From ELISA experiment (Fig. 1), normal sera showed a mean O.D: 0.175 from triplicates, However sera from Grade 1: O.D is 0.25, Grade 2: O.D is 0.46, and Grade 3: O.D is 0.52 that showed increased expression of the antibody reactive H11 antigen. Even though eight samples from 18 different cancers show H11 antigen activity with UNIVmAb, similar results were also observed with the remaining cancer samples.

### UNIVmAb reactive H11 proteins were overexpressed in human cancer serum shown in Fig. 2

**Fig. 2:**
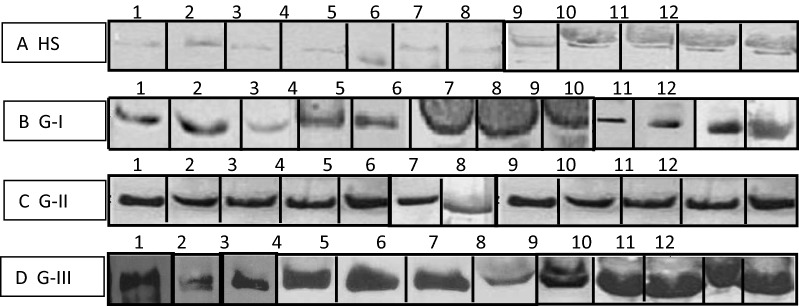
UNIVmAb reactive human sera antigen expression by western blot. **a** Healthy controls serum from 12 different individuals. **b**–**d**: Grade 1, Grade 2, and Grade 3. Lanes 1 and 2: Tongue, Lanes 3 and 4: Salivary, Lanes 5 and 6: Breast, Lanes 7 and 8: Colon, Lanes 9 and 10: Cervix, Lanes 11 and 12: Ovary

**Fig. 3:**
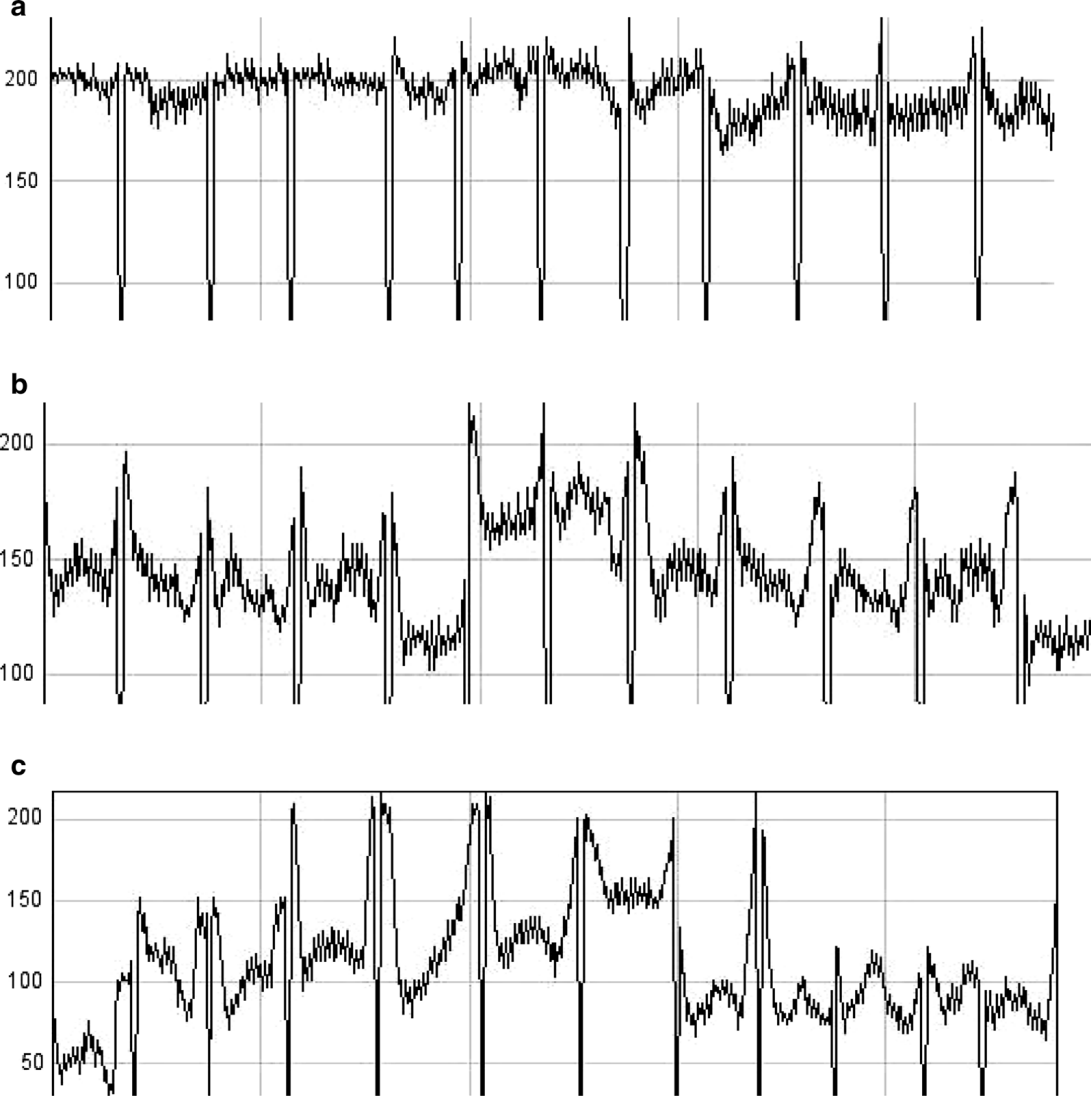
Scion image analysis, Normal (**a**) Grade 2 (**b**) and Grade 3 (**c**). Scion Image Analysis of Panel 1: Scion image clearly showed the overexpression of H11 antigen as the tumour progress to grade three. However, we showed after Western blot analysis that UNIVmAb reacted antigen overexpression is real and accepted when tested with all other cancers sera samples

To detect whether UNIVmAb reactive antigens were present in circulation, we conducted western blot analysis of normal and various cancers sera samples using UNIVmAb. The UNIVmAb showed reduced H11 reactivity in normal sera at 57 kDa (Fig. 2 a), the intensity of reaction is enhanced in tumour samples of high-grade cancers (Fig. 2d vs b, c). We compared this with 70 normal samples and 50 cancer patient’s samples of different grade. We have shown H11 expression in 12 different healthy individuals sera samples in Fig. 2 (panel a lanes 1 to 12). The UNIVmAb reaction with serum proteins of six different cancer sera of different cancer patients are shown in panel b, c and d. The presence of UNIVmAb reactive protein in normal and eighteen cancer subjects suggests that H11 protein might have several biological functions. Similar results were observed in the tumour/normal tissues [13]. Figure 3 Scion image analysis of the western blot showed that UNIVmAb reacted antigen overexpression is real and accepted when tested with all other cancers sera samples.

Western blot analysis with bHA probe showed that the detected serum HABP expression is H11 antigen (Fig not shown) and HA-Oligo competition showed 80% reduction of antigen expression in all cancer’s sera. These results indicate that the 57 kD, named H11 antigen is a hyaluronan binding protein (data not shown). We performed immunoprecipitation and affinity purification of normal and cancer patient’s serum proteins with mAb to prove that mAb is reacting with 57 kDa protein with high specificity. As expected the normal sera showed low levels of circulatory 57 kDa by western blot, whereas, advanced cancer sera showed overexpression of 57 kDa antigen. To show that the cancer antigen 57 kDa is associated with carrier protein such as Albumin we used Cibacron blue gel exclusion method. Eluted fractions were tested for reactivity with b-HA and UNIVmAb, showing a strong reaction at 57 kDa (data not shown). To investigate UNIVmAb reactive cancer antigen may be related to a known hyaladherin, CD44 (std); Whether, UNIVmAb immunoprecipitated protein can pull down CD44 reactive protein, we used stomach grade lll cancer sample, which overexpressed for mAb reacted H11 antigen and normal sera were tested. We find no reaction to HCAM antibody to the H11 antigen (data not shown).

## Conclusion

Blood-borne metastasis is the greatest obstacle to cure patients with cancers. The abundant blood proteins, such as albumin, immunoglobulin, etc.; may mask the less abundant proteins, which are usually potential markers. There are many known specific serum markers but not a common biomarker for various cancers sera.

UNIVmAb detected the overexpression of serum specific H11 antigen in various cancer’s sera, offers significant advantages. In the present study, screening serum samples from 70 normal and 50 cancer patients samples (18 different cancer types of various grade) of different grades we predicted that the UNIVmAb might be used as a antibody to detect a common cancer biomarker. This Mab detected a protein H11 and is unique HABP not identified earlier in serum and serum albumin may be the carrier protein of H11 antigen. Still unanswered question is, what is the nature of 57 kDa H11 antigen. We investigated the H11 antigen by proteomic analysis from grade ll adenocarcinoma of the colon. We observed the presence of IgGH1, member of immunoglobulin super family (data not shown). The present data showed that UNIVmAb detected the H11 might be a unique hyaladherin and can be used as a common biomarker for progressive human cancers sera.

## Limitations

At present, there is no common biomarker for multiple cancer detection. The present study could reflect the UNIVmAb reactive serum antigen as a common biomarker for multiple cancers. In addition, future experiment on the proteomic analysis will help us identify the nature of the antigen as a potential common biomarker.

## Data Availability

All data generated or analyzed during this study are included in this published article.

## Abbreviations

HA: hyaluronan
HABP: hyaluronan binding protein
b-HA: biotinylated hyaluronan
HA-oligo: hyaluronan oligosaccharides
ABTS: 2.2′-azino-Bis-3-ethylbenzothiazoline-6-sulfonic acid
